# Automated System for Kinetic Analysis of Particle Size Distributions for Pharmaceutically Relevant Systems

**DOI:** 10.1155/2014/810589

**Published:** 2014-07-16

**Authors:** John-Bruce D. Green, Phillip W. Carter, Yingqing Zhang, Dipa Patel, Priyanka Kotha, Thomas Gonyon

**Affiliations:** Baxter Healthcare, Technology Resources, 25212 W. Illinois Route 120, Round Lake, IL 60073, USA

## Abstract

Detailing the kinetics of particle formation for pharmaceutically relevant solutions is challenging, especially when considering the combination of formulations, containers, and timescales of clinical importance. This paper describes a method for using commercial software Automate with a stream-selector valve capable of sampling container solutions from within an environmental chamber. The tool was built to monitor changes in particle size distributions via instrumental particle counters but can be adapted to other solution-based sensors. The tool and methodology were demonstrated to be highly effective for measuring dynamic changes in emulsion globule distributions as a function of storage and mixing conditions important for parenteral nutrition. Higher levels of agitation induced the fastest growth of large globules (≥5 *μ*m) while the gentler conditions actually showed a decrease in the number of these large globules. The same methodology recorded calcium phosphate precipitation kinetics as a function of [Ca^2+^] and pH. This automated system is readily adaptable to a wide range of pharmaceutically relevant systems where the particle size is expected to vary with time. This instrumentation can dramatically reduce the time and resources needed to probe complex formulation issues while providing new insights for monitoring the kinetics as a function of key variables.

## 1. Introduction

Automated approaches to pharmaceutical solution preparations are gaining in popularity due to reductions in sample preparation errors [1]. Automation of analytical investigations of drug solutions is improving the throughput in degradation analysis [2] and driving efficiencies in bioanalytical assays [3] as well. Developing automated approaches for measuring changes in solution particle concentrations is important because solution particle characteristics are often a key indicator of product quality and safety. Light obscuration based instrumental particle counting/sizing techniques are widely used in the pharmaceutical industry to determine the concentrations and sizes of particles that are present in parenteral solutions. Examples include counting and sizing of subvisible particulate matter per USP Chapter 〈788〉 [4] or per European Pharmacopoeia 2.9.19 [5] and measuring the concentrations of larger sized globules in nutritional emulsions per USP Chapter 〈729〉 [6]. Emerging regulatory requirements are further increasing scrutiny on protein-based formulations and the need for understanding protein aggregation kinetics including large particle formation [7]. Having appropriate, automated tools available will help meet the greater demand for solution particle characterization being driven by regulatory requirements.

The typical instrumental particle counting analyses include periodic testing over long-term storage conditions by trained analysts with calibrated, prequalified equipment to verify parenteral products are within specified particulate matter requirements for their documented shelf life. The long-term storage conditions of the test articles are typically several months or years in duration and may include evaluating various sample configurations at multiple targeted temperatures and levels of humidity. While several autosamplers are commercially available to augment the testing efficiency of the instrumental particle counting analyses, the commercial particle counting autosamplers generally require the analysts to transfer portions of each test article solution from its original container to a separate particle-free container which is then placed onto an automated conveyor to deliver the sample to the particle counting liquid sampler. The ability to use commercial autosamplers to complete USP Chapter 〈788〉 light obscuration particle testing of products directly from clinically relevant containers is limited to one example of the use of a robotic sample handling device [8, 9]. In addition, the ability to continuously add formulation components to multiple test articles stored in pharmaceutically relevant packaging or to apply physical stress to samples while monitoring the particle size distribution continuously over a short time period such as 24 hours is not easily performed with the commercial particle autosamplers. Utilizing trained analysts as resources to execute all the measurements raises the experimental costs significantly and presents logistic challenges as well. Thus, there is clearly a need for improving the tools available to study the time-dependent behavior of parenteral solutions especially on the time scales between product activation and injection into the patient. This time scale is referred to as a short-term stability regime and typically ranges from hours to several days in duration and may require temperature control from refrigeration (5°C) to body temperature (37°C).

The instrumental particle counter is a very useful tool to monitor such short-term stability of parenteral solutions to verify formulation integrity and performance by monitoring particle size distribution kinetics. Typical clinical use of these formulations can occur over a 24-hour period representing a useful time frame for continuous monitoring. One example to consider is the evolution of emulsion droplet size as a function of storage, stress, and time. USP Chapter 〈729〉 specifies the use of a particle counter to examine the volume weight percentage of droplets greater than 5 *μ*m (PFAT_5_) in parenteral emulsions as one of the two assays to characterize the globule size distribution of the emulsion. As emulsions are destabilized, there can be an increase in observed fraction of large globule sizes as physical changes occur to the oil droplets. The ability to automate the PFAT_5_ analysis to sample continuously and directly from multiple containers can provide unique insight into the kinetics of evolution of the emulsion globule size distribution for parenteral total nutrient admixtures (TNA) which cannot be inferred from two-point measurements.

Another area of industrial interest involves the optimization of formulation conditions. Formulation composition, for example, amino acid, dextrose, electrolyte, calcium, and phosphate concentrations, can have a profound impact on the kinetics of precipitation in different parenteral solutions. Calcium and/or phosphate are routinely added to parenteral admixtures containing amino acids and dextrose to meet patient needs which may increase the risk of precipitation of calcium phosphate. USP Chapter 〈788〉 specifies the use of a light obscuration particle counter as one method to determine the particle burden of parenteral solutions. Kinetic analysis of precipitate size distributions can provide unique insight into calcium phosphate compatibility.

In both of these examples, the storage interval would be considered short-term, generally less than 7 days and, in some cases, as short as a few hours in duration. Ideally, in order to obtain kinetic information on a short-term basis studying the dynamic nature of changing particle populations, it is preferred to monitor the particle size distribution in real time in actual containers used in clinical settings. While these samples could still be measured manually by trained analysts, this would require multiple analysts to be readily available throughout the short-term test condition to complete the testing at the desired multiple time points. For a seven-day test, this would be a minimum of 168 hours of analyst coverage assuming the laboratory was safely available for an individual throughout the study. In addition, for overcoming cost and constraints due to manpower issues, an automated particle sizing/counting tool offers the following advantages:

- reduction of sample handling errors, for example, contamination or different volumes;
- simultaneous analysis of multiple samples;
- ability to control environmental conditions, for example, temperature, humidity, and so forth;
- flexibility to acquire test results at intervals ranging from minutes to days;
- hands-free automation with remote view.

The goal of this work is to demonstrate an improved efficiency and an ability to gain insight into the kinetics of pharmaceutically relevant particle phenomena. These improvements are accomplished by augmenting commercial particle counting/sizing tools with automation software and a multiport injection valve to allow for short-term monitoring of relevant formulations.

## 2. Materials and Methods

### 2.1. Sensitivity of Nutritional Emulsions to Agitation

#### 2.1.1. Preparation

This study probed the emulsion stability of a total nutrient admixture (TNA) by examining how agitation affected the kinetics of PFAT_5_. Three emulsion admixtures were sampled directly from ethylene-vinyl acetate (EVA) containers via an autodiluting AccuSizer every 90 minutes over the course of 24 hours and at 3 different levels of agitation.

The nutritional admixture was prepared by mixing 1 part lipid emulsion (20% ClinOleic, code ADB9501, Baxter, Maurepas-Cedex, France) with 8 parts amino acid/dextrose solution (CLINIMIX E 5/25, code 2B7723, Baxter, Deerfield, IL). ClinOleic is an oil in water emulsion in which the oil composition is 20% soybean oil and 80% olive oil [10], and CLINIMIX E 5/25 is a sterile, nonpyrogenic, hypertonic, and dual-chamber product that when mixed produces a final amino acid concentration of 5% and a final dextrose concentration of 25% and contains additional electrolytes [11]. Nine separate 1L ethylene-vinyl acetate (EVA) containers (code 2B8114, Baxter, Deerfield, IL) were each filled with approximately 900 g of the TNA solution. Each container was fitted with a polyethylene catheter (PE200, B&D Development, Inc., Franklin Lakes, NJ), thereby enabling direct sampling from the sealed containers.

The TNA filled EVA containers were exposed to three different agitation levels: resting versus gentle rocking versus continuous vigorous shaking. One laboratory shaker (Catalog 12620-910, VWR, Radnor, PA) with setting at 1 RPM was used to gently rock three samples while a plate shaker (Catalog VXR Basic, IKA Works Inc., Wilmington, N.C.) with setting at 100 RPM was used to continuously agitate three other samples in a much more vigorous manner.

PFAT_5_ measurements were performed directly from the EVA containers with an AccuSizer model 780 automatic particle sizer (APS) (Particle Sizing Systems, Santa Barbara, CA) with a previously calibrated LE 400 sensor using L2W788 software, version 2.19. The APS sample valve was used to deliver sample volumes to the APS for testing. Information regarding the experimental conditions is presented in Table 1.

#### 2.1.2. Automation

The PSS APS 780 autodilutor is designed to deliver and dilute a fixed volume of a concentrated emulsion or suspension to a light obscuration sensor for particle/globule counting and sizing. The APS autodilutor is controlled by the AccuSizer APS software and the test sample may be delivered to the fixed volume loop prior to the measurement either with the built-in peristaltic pump or with a syringe manually by an analyst prior to the measurement. After the fixed volume loop is filled, the contents of the loop are delivered by the APS autodiluter to a primary dilution chamber where it is mixed with filtered diluent and then a portion of that diluted solution is then mixed with more filtered diluent to lower the concentration of globules/particles to the desired countable level. Finally, the diluted sample is delivered by the APS to the light obscuration sensor at the desired flow rate for sizing and counting. After completion of the measurement, the APS will automatically rinse the primary dilution chamber and sensor path to prepare for the next injection.

The nine samples were connected to the APS autodiluter via a 10-port stream-selector valve (Catalog C25-6180E, Valco Instruments, Houston, TX). The 10-port valve was controlled by the valve application software (VCOM, Valco Instruments, Houston, TX) and allowed the nine samples to be sequentially delivered to the APS autodiluter for subsequent globule size distribution measurements. The AccuSizer and the valve were both controlled with the automation software such that the TNAs were sampled periodically over approximately 24 h, about once every 90 minutes, to determine the concentration of PFAT_5_ at ambient conditions.

In a conventional measurement, the APS autodiluter requires an analyst to be present at the start of each measurement to manually switch between any samples that need to be tested and to enter the necessary keyboard and mouse commands to complete a measurement. The ability to automatically switch between the AccuSizer APS program and the valve program and to automatically click on the desired action buttons in each program unattended in the correct order to complete a measurement was of paramount importance.

Activation and control of the AccuSizer instrument and sampling valve was performed with automation software (Automate 7, Network Automation, Los Angeles, CA) which is a productivity software tool that can be easily programmed by the user to open multiple programs and enter desired keyboard and mouse actions and repeat as needed [12, 13]. While Automate 7 was used for this study, there are other alternative scriptable desktop automation tools such as AutoHotKey [14] which could also have been used.

**Figure 1:**
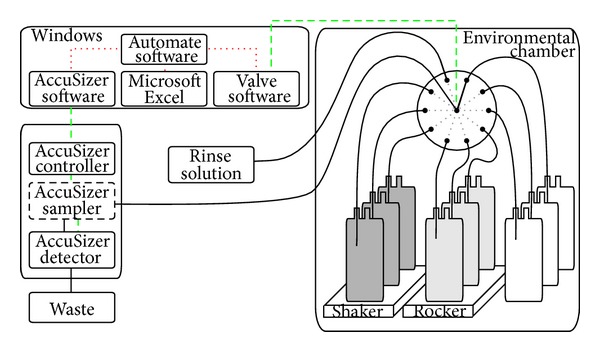
A schematic of the automated system is shown. The narrowly spaced red dashed lines represent the Automate software control of key Windows applications. The dashed green line represents the electronic control of instrumentation, and the solid black lines represent fluid paths. The thick solid lines represent the fluid path of the test samples from a single sample container through the 10-port valve to the AccuSizer system and then finally to waste.

The normal order of operation was to first open the valve software to select the appropriate valve port and retrieve needed sample information entered into Microsoft Excel, followed by the AccuSizer software to perform the measurement. After the measurement was completed, the Automate program switched back to the valve software to select the appropriate port for the next sample to be tested and repeated as necessary. A schematic of the setup for the automated AccuSizer APS experiment is shown in Figure 1.

### 2.2. Precipitation from Nutritional Formulas

#### 2.2.1. Preparation

The base formulation used in the calcium phosphate solubility evaluation portion of this study was comprised of an amino acid/dextrose parenteral nutrition solution in a flexible dual chamber container (CLINIMIX 5/15, code 2B7709, Baxter, Deerfield, IL). After activating the peel-seal between the dual chambers and thoroughly mixing the container, the resulting solution has a final amino acid concentration of 5% and a dextrose concentration of 15% [15].

Two experiments were performed with an AccuSizer syringe sampler to measure the particle size distribution at regular intervals for extended times to probe the extent of calcium phosphate precipitation. The first experiment focused on the kinetics of calcium phosphate precipitation for CLINIMIX 5/15 admixtures with various levels of added calcium. The latter experiment examined the kinetics of precipitation for several pH-adjusted CLINIMIX 5/15 admixtures as calcium was infused into the samples.

In the initial experiment, as presented in Table 2, the automated particle counter with sampling valve was used to assess the effect of a bolus addition of 0.232 mmol/mL or 0.465 mEq/mL calcium gluconate (code C311B1, PPC, Canada) to CLINIMIX 5/15. The calcium additions produced eight different CLINIMIX formulations, with the following calcium concentrations (i.e., 0.9, 3.9, 5.3, 6.4, 7.3, 8.3, 9.8, and 12.7 mmol/L Ca^2+^). The phosphate concentration for all the eight was fixed at 24 mmol/L to compare the induction times and rates of precipitation of calcium phosphate during the initial 100 hours of storage at 37°C.

The latter experiment, as presented in Table 3, probed the effect of the continuous addition (1.5 mL/hour for 24 hours) of calcium gluconate to CLINIMIX 5/15 solutions. Using an eight-channel syringe pump, calcium gluconate was infused into eight pH-adjusted CLINIMIX 5/15 solutions ranging from pH 4.5–8.0 (i.e., 4.5, 5.0, 5.5, 6.0, 6.5, 7.0, 7.5, and 8.0). The phosphate and calcium levels were the same for all eight samples, so this experiment highlights how the induction times and precipitation rates depended upon the formulation pH.

All test articles were prepared in a laminar flow hood using glass bottles that were precleaned by exhaustively rinsing with filtered distilled water prior to filling with CLINIMIX 5/15 solutions. The order of addition was as follows: parenteral nutrition solution, sterile water for injection (code 2B0304, Baxter, Deerfield, IL, USA), 4 mmol/mL sodium chloride solution (code 2900-25, American Regent, Shirley, NY, USA), 2 mmol/mL potassium chloride solution (code 96520, APP, Schaumburg, IL, USA), 3 mmol/mL potassium phosphates (code 2350-25, American Regent, Shirley, NY, USA), and 2 mmol/mL magnesium sulfate solution (code 5491, Sandoz, Canada). A magnetic stir bar was added to each bottle to promote adequate mixing with continuous stirring at 200 rpm. The bottles were transferred to the environmental chamber and each connected to the 10-port sampling valve to allow for periodic particulate counting testing. If necessary, pH adjustment was performed with either 1 N NaOH or 1 N HCl to achieve targeted pH values. All units were mixed by inverting the glass bottles 5 times prior to placing the glass bottles into the environmentally controlled storage chambers.

#### 2.2.2. Automation

The automated AccuSizer 780 syringe injection sampler (SIS) testing was facilitated by the same equipment listed in the emulsion experiments above. As before, this design allowed direct sampling from the containers. Particle counting of each formulation was determined using an AccuSizer model 780 counter with SIS (syringe injection sampler) utilizing a previously calibrated LE 400 sensor in extinction mode. Four sequential 3 mL aliquots from each test solution were evaluated with the AccuSizer particle counter determining the cumulative particle counts/mL greater than or equal to the following sizes: 1.5, 2.0, 3.0, 4.0, 6.0, 8.0, 10.0, 15.0, 25.0, and 50.0 *µ*m. The results from the first 3 mL aliquot test from each sample were excluded from the average of the last three aliquots. The AccuSizer SIS is designed to deliver and test a fixed volume of a solution and it is controlled by the proprietary software, using a built-in syringe to deliver the sample to be measured at the desired flow rate to the light obscuration sensor. After completion of particle counting, the SIS may be manually rinsed with particle-free water to prepare for the next injection. In this configuration, a 10-port valve was placed in line with the SIS sampler to allow for automatic selection of multiple samples. Activation and control of the SIS instrument and sampling valve was performed with automation software (Automate 7, Network Automation, Los Angeles, CA) in similar manner as described above.

Various amounts of 0.232 mmol/mL calcium gluconate were added as bolus injections into the glass bottles after the 5th run on the particle counter was completed, approximately 2.5 hours after starting the particle counter. Samples with concentrations that demonstrated excessive visual precipitation (e.g., 12.7 mmol/L [Ca] with 24 mmol/L [P] or pH 8.0) during the course of testing were removed from testing after precipitation occurred to eliminate possibility of sensor clogging.

## 3. Results and Discussion

### 3.1. Sensitivity of Nutritional Emulsions to Agitation

**Figure 2:**
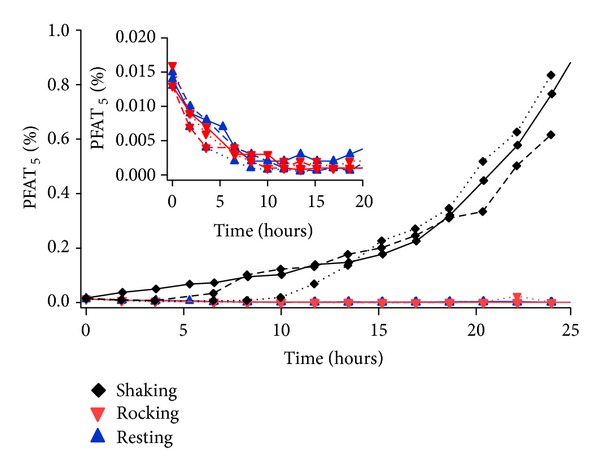
The effects of three different agitation levels on emulsion stability were evaluated in triplicate over a 24-hour period by measuring the changes PFAT_5_ at rest (upward triangle), gently rocked (downward triangle), and vigorously shaken (diamond). The inset focuses on the rest and gentle rock conditions where PFAT_5_ is observed to decrease significantly especially at early times.

An emulsion, consisting of a dispersion of oil and water phases, is a poised thermodynamic system and is considered to be inherently unstable. A variety of factors including but not limited to pH, temperature, emulsifier, oil composition, oil concentration, and time can be used to destabilize parenteral nutritional emulsions causing the emulsion's physical attributes (i.e., appearance, globule size distribution, zeta potential, etc.) to change [16]. Destabilization of emulsions can be segmented into 4 classes, flocculation, creaming, coalescence, and Ostwald ripening. In all 4 classes of destabilization, a change in the measured larger sized globules may be observed over time as the emulsion becomes more and more unstable. In this study, the effect of mechanical agitation on the observed globule size distribution of a nutritional admixture was examined over approximately 24 hours as shown by the time-dependent behavior of PFAT_5_ as a function of agitation in Figure 2.

Figure 2 compares the PFAT_5_ levels of the TNAs stored in the 1 L EVA containers and exposed to various agitation conditions (e.g., resting versus gentle rocking versus continuous shaking) for 24 hours. The initial PFAT_5_ levels for all samples were approximately 0.015. Elevated PFAT_5_ results (~0.20 and higher) were observed for the shaking samples compared to the resting and gentle rocking samples (~0.005), particularly after 15 hours of continuous shaking. In the initial 10 hours, there was considerable variability between the three replicates of the shaking units in terms of when the PFAT_5_ started to increase above the initial values of ~0.015. For all shaking samples after 10 hours, the PFAT_5_ values continually increased to the 24-hour endpoint reaching values as high as 0.90 at the 24-hour interval for one sample. There is fairly good reproducibility between replicates in the time period from 15 to 24 hours.

In contrast to the results for shaking samples, the inset at the top of Figure 2 shows a consistent decrease in PFAT_5_ from an initial value of 0.015 for both the resting and the gentle rocking samples to values below 0.005. In particular, the initial 10 hours of the study show the least variation between large globule profiles represented by PFAT_5_. The decrease in PFAT_5_ over time present in the resting and rocking samples has been observed previously [17] and is attributed to triglycerides being absorbed to the EVA container. While the mechanistic details are not clear, the larger oil globule droplets are thought to preferentially and irreversibly attach to the surface of the EVA container decreasing their solution concentration over time.

In a clearly differentiated manner, the PFAT_5_ of the shaking samples was observed to increase over the 24-hour test interval. The mechanism for destabilization of the emulsion by mechanical agitation followed by an increase in globule size as observed for the shaken samples is unclear, although the use of mechanical stress to negatively impact the stability of emulsions has been cited previously [18]. It is possible that the mechanical agitation caused multiple droplets to fuse together to form larger droplets consistent with coalescence mode of destabilization. Perhaps this is accelerated at the air-water interface where the shaking samples have significant air bubble formation in contrast to the other samples.

Of additional significance is the great variation in PFAT_5_ evolution that occurs during the first ten hours of shaking. In fact, the relative PFAT_5_ values for the three replicates before 12 hours are not retained at 24 hours. This does not appear to be a nucleated event where some initial small fraction of globule growth creates a downhill cascade of more rapid growth. Additionally, it is expected that the same mechanism of large globule removal to the EVA container is occurring simultaneously. Thus, the observed increase in PFAT_5_ is the sum of shear-induced large globule formation increases and globule adsorption to container decreases.

The time-dependent globule size data on the multiple nutritional admixture samples was easily generated over the approximately 24-hour sampling time using the automated tool. Future testing may help identify the key characteristics for shear-induced emulsion droplet growth such as shear rate, temperature, and role of air bubbles. Mitigating such growth would have clinical significance so as to minimize the injection of large globules to patients, thereby improving the safe delivery of parenteral emulsion admixtures.

### 3.2. Precipitation from Nutritional Formulas

Addition of calcium to phosphate containing amino acid/dextrose parenteral nutrition solutions can result in the precipitation of calcium phosphate crystals under certain conditions. The solubility of calcium phosphate in nutritional parenteral admixtures has been discussed extensively [19–21] with general trends associated with the degree of supersaturation:

- precipitation is less likely at lower calcium and phosphate concentrations;
- precipitation is less likely to occur at lower pH.

Increased concentrations of calcium in phosphate containing solutions are more likely to precipitate as more calcium is available to bind with the available dibasic phosphate in a high concentration calcium formulation. Probability-based calcium phosphate stability curves have been discussed recently [22] and are based on solution measurements made at a single, fixed time. It is instructive to look at the kinetics of the process as well.

**Figure 3:**
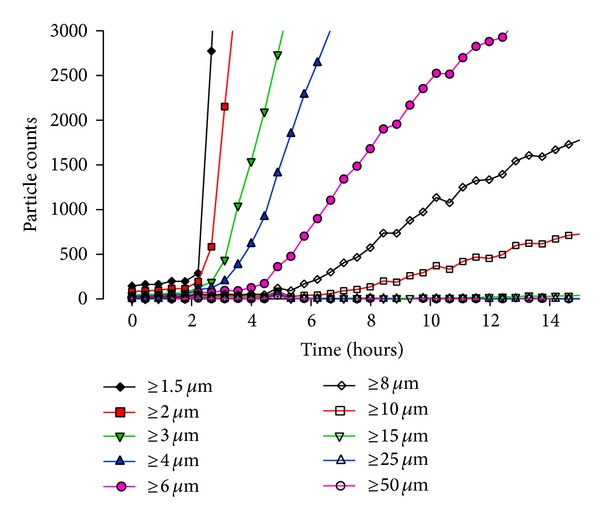
The particle size data versus time for a 15-hour period presenting the cumulative counts at various sizes including ≥1.5 *µ*m (diamonds), ≥2.0 *µ*m (squares), ≥3.0 *µ*m (downward triangles), ≥4.0 *µ*m (upward triangles), ≥6.0 *µ*m (circles), ≥8.0 *µ*m (empty diamonds), ≥10.0 *µ*m (empty squares), ≥15 *µ*m (empty downward triangles), ≥25 *µ*m (empty upward triangles), and ≥50 *µ*m (empty circles), for the 12.7 mmol/L [Ca^2+^] sample.

The size-dependent increase in particle counts/mL over a 15-hour time period for the highest calcium concentration, 12.7 mmol/L, tested is presented in Figure 3.

**Figure 4:**
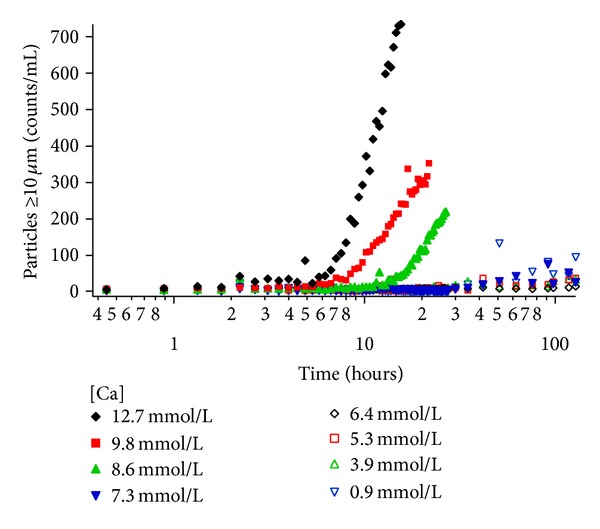
The effects of calcium concentration ranging from 0.9 mmol/L to 12.7 mmol/L on calcium phosphate particle formation for a nutritional admixture stored at 37°C are monitored through the appearance of particles ≥10 microns in size. The time-dependent evolution of particle counts/mL shows shorter induction times and greater particle concentrations with increasing calcium concentrations greater than 7.3 mmol/L.

The highest counts were observed in the smallest sizes first and then increases in the larger sizes were observed later. The calcium phosphate particle formation kinetics as a function of calcium concentration for a 2-in-1 admixture is depicted in Figure 4. While only the cumulative counts/mL ≥10 *μ*m are shown in Figure 4, the other cumulative sizes analyzed showed the same general trend as a function of calcium.

The results show an increase in particle counts/mL over time for the higher calcium concentrations (12.7, 9.8, and 8.3 mmol/L) after the bolus of calcium gluconate was added to each bottle at ~2.5 hours. For these elevated calcium concentrations, higher particle counts/mL were observed with increasing calcium concentration. For the eight amino acid formulations with varying calcium concentrations, as the calcium concentration decreased from 12.7 to 8.3 mmol/L, the induction time to precipitation increased. Once initiated, the rate of cumulative particle counts/mL increased with increasing calcium concentration. The lower calcium concentrations (7.3 mmol/L and below) did not generate any significant increase in particle counts/mL. Figure 4 clearly shows the span of kinetic data accessible from the automated tool where unique induction times, growth rates, and final particle counts/mL can be simultaneously obtained.

**Figure 5:**
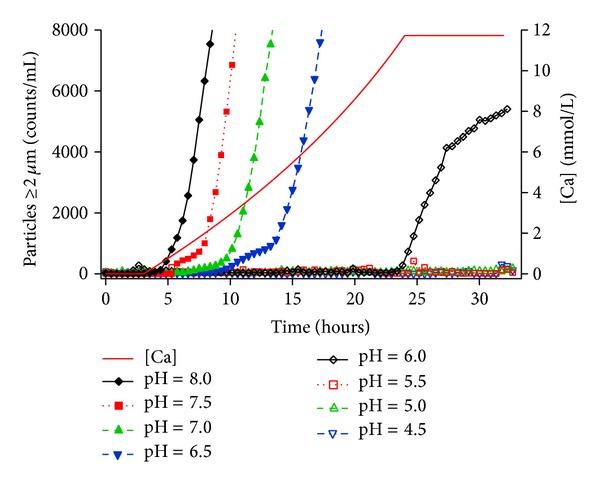
The pH dependence ranging from 4.5 to 8.0 of calcium phosphate particle formation ≥2 microns for a nutritional admixture stored at 37°C is monitored with simultaneous calcium addition over a 30-hour period. The concentration of calcium started at 0 mmol/L initially and reached ~12 mmol/L after 24 hours of continuous addition while the concentration of phosphates was ~24 mmol/L throughout the experiment. For pH ≥ 6, as the pH increases, the calcium addition time is shorter before the appearance of increased particle counts/mL. There are also observed inflection points in the rates of increase in particle concentrations for the admixtures where calcium phosphate precipitation was observed.

An additional titration approach to measuring formulation compatibility was performed by adding calcium to a CLINIMIX admixture solution. The number of particles/mL ≥2 *μ*m for the continuous infusion of calcium into the pH-adjusted CLINIMIX samples is shown in Figure 5.

Only the cumulative counts/mL ≥2 *μ*m are shown as the other sizes collected showed the same general trend. The addition of calcium started at ~3.5 hours and was continually added for 24 hours to constantly increase the calcium concentration to 12 mmol/L where it remained for the duration of the experiment as shown in Figure 5. Increases in particle counts/mL were observed for solutions where the pH ≥ 6 but not for the pH < 6 range. The induction time to particle formation was inversely related to pH values with the higher pH samples generating particles with shorter induction times. The particle counts/mL of the pH 6.0 formula increased last. In addition, the pH 6.0 sample had the lowest increase in particle counts/mL of the samples that had an increase in particle counts/mL.

As the pH of most nutritional parenteral solutions is between 4 and 8, understanding the pH-dependent behavior of the two forms of calcium phosphate, monobasic calcium phosphate Ca(H_2_PO_4_)_2_ and dibasic calcium phosphate CaHPO_4_, is critical to evaluating and predicting the probability of precipitation of calcium phosphate crystals [23–25]. As described elsewhere [26], as the pH approaches 7.2, which is the pKa of phosphoric acid, the concentration of the dibasic form of phosphates increases and precipitation is more likely to occur due to the relatively low solubility (e.g., 0.3 g per L at 25°C) of this form of calcium phosphate. As the pH decreases further below 7.2, the monobasic form of calcium phosphate becomes more prevalent and the risk of precipitation decreases as the solubility of the monobasic form (e.g., 18 g per L at 25°C) is much higher than the dibasic form. In Figure 5, the solutions with pH values below 6.0 did not exhibit increased particle counts/mL while the samples formulated at pH at 6.0 and above did exhibit increased particle counts/mL which is consistent with the pH-dependent rule.

The time-dependent behavior observed in these datasets clearly demonstrates the utility of this automated tool comprised of a calibrated particle counter, automation software, and a multiport injection valve to study the kinetic analysis of relevant systems.

## 4. Conclusions

The use of Automate software with a multiport sampling valve allowed two different instrumental particle counters to sample directly and continuously multiple relevant containers to study the dynamic nature of changing particle populations. The PFAT_5_ of TNA was evaluated continuously with direct sampling from pharmaceutically relevant containers comparing three different types of agitation (e.g., rest versus continuous shaking versus gentle rocking). The results showed an increase in PFAT_5_ for the most vigorous shaking and a decrease in PFAT_5_ for containers at rest and containers with gentle shaking. The automated system was also demonstrated to be useful in the evaluation of calcium phosphate particle formation for two distinct experiments with 5% amino acid/15% dextrose admixtures. In the first experiment, the effect of calcium concentration on a 5% amino acid/15% dextrose admixture was studied and showed precipitation occurring with higher calcium concentrations above 8.3 mmol/L. As the calcium concentration decreased from 12.7 to 8.3 mmol/L, the induction time to precipitation increased, and, once precipitation began, the rate of precipitate generation decreased. In the second application, the automated system was used to study the effect of varying pH of a 5% amino acid/15% dextrose admixture and showed precipitation occurring fastest at higher pHs with no precipitation observed below pH 6.0.

## Figures and Tables

**Table 1:** Conditions for probing parenteral emulsion dynamics—agitation experiment.

Parameter	Setting
Test article	3-in-1 total nutrient admixture-ClinOleic with CLINIMIX E 5/15
Agitation	Rest versus continuous rocking versus shaking
Container	1-liter EVA
Measurement	PFAT_5_ (%) with APS
Interval testing	Continuous for 24 hours, approximately every 90 minutes
Storage condition	Ambient

**Table 2:** Calcium phosphate solubility in an admixture—conditions for effect of [Ca^2+^].

Parameter	Setting
Test article	5% amino acid/15% dextrose with electrolytes
Container	1-liter glass bottle
Temperature (°C)	37
Calcium (mmol/L)	0.9, 3.9, 5.3, 6.4, 7.3, 8.3, 9.8, and 12.7
Phosphates (mmol/L)	24
Threshold particle size (*μ*m)	≥1.5, 2.0, 3.0, 4.0, 6.0, 8.0, 10.0, 15.0, 25.0, and 50.0
Calcium addition	After 2.5 hours, bolus injection to achieve [Ca^2+^] above
Interval testing	Periodically for approximately 100 hours, approximately every 30 minutes

**Table 3:** Calcium phosphate solubility in an admixture—conditions for effect of pH.

Parameter	Setting
Test article	5% amino acid/15% dextrose with electrolytes
Container	1-liter glass bottle
Temperature (°C)	37
Targeted pH	4.5, 5.0, 5.5, 6.0, 6.5, 7.0, 7.5, and 8.0
Threshold particle size (*μ*m)	≥1.5, 2.0, 3.0, 4.0, 6.0, 8.0, 10.0, 15.0, 25.0, and 50.0
Calcium (mmol/L)	0.232 mmol/mL [Ca^2+^] added at 1.5 mL/hour to 12 mmol/L
Phosphates (mmol/L)	24
Interval testing	Periodically for approximately 35 hours, approximately every 20 minutes
